# Retraction: Suppression of uPAR retards radiation-induced invasion and migration mediated by integrin β1/FAK signaling in medulloblastoma

**DOI:** 10.1371/journal.pone.0328091

**Published:** 2025-07-15

**Authors:** The PLOS One Editors

After the publication of this article [1], concerns were raised about the results presented in Figs 2, 4, 6–8, and S3. Specifically,

- The following panels appear similar:
  - Fig 2D (left) N-Cadherin and Fig 2D (right) Integrin β-1
  - Fig 4A DAOY GAPDH and Fig 4A. D.283 GAPDH
  - Fig 4B DAOY GAPDH and Fig 4B. D.283 GAPDH
  - Fig 7A DAOY Integrin β1 of this article [1] and Fig 5A SF3061 uPA of [2, corrected in 3]
- The following panels appear to partially overlap:
  - Fig 6A DAOY Non IR Con and Fig 6A DAOY Non IR pSV
  - Fig 8A Non-IR pU and Fig 8C Non-IR pSV
  - Fig S3 pFAK(Y397) Non-IR Control, Fig S3 pFAK(Y397) IR Control, and Fig S3 pFAK(Y397) IR pSV.

The authors did not respond to the journal’s communications.

In light of the above concerns, which call into question the reliability and integrity of the published image data and their associated quantified results, the *PLOS One* Editors retract this article.

All authors either did not respond directly or could not be reached.
